# Recognition and Treatment of BCG Failure in Bladder Cancer

**DOI:** 10.1100/tsw.2011.30

**Published:** 2011-03-07

**Authors:** Andrew J. Lightfoot, Henry M. Rosevear, Michael A. O'Donnell

**Affiliations:** Department of Urology, University of Iowa, Iowa City, USA

**Keywords:** bladder cancer, BCG, metastasis, disease progression, treatment failure, neoplasm recurrence

## Abstract

Patients with high-grade Ta, T1, or carcinoma *in situ* non–muscle-invasive bladder cancer (NMIBC) are at high risk for recurrence and, more importantly, progression. Thus, both the American Urological Association and European Association of Urology recommend initial intravesical treatment with bacillus Calmette-Guerin(BCG) followed by maintenance therapy for a minimum of 1 year. The complete response rate to BCG therapy in patients with high-risk NMIBC can be as high as ∼80%; however, most patients with high-risk disease suffer from recurrence. BCG failure can be further characterized into BCG refractory, BCG resistant, BCG relapsing, and BCG intolerant. Current recommendations include one further course of BCG or cystectomy. In patients who continue to fail conservative treatment and who refuse surgical therapy or are not surgical candidates, treatment options become even more complicated. In this setting, treatment options are limited and include repeat BCG treatment, an alternate immunotherapy regimen, chemotherapy, or device-assisted therapy. To date, however, further research is necessary for all secondary treatment options in order to determine which might be the most efficacious. All conservative treatments should be considered investigational. Currently, cystectomy remains the standard of care for high-risk patients who have failed BCG therapy.

## INTRODUCTION

Approximately 80% of all newly diagnosed bladder cancers are found to be non–muscle invasive[1,2]. Non–muscle-invasive bladder cancer (NMIBC) is defined as a tumor that invades up to the lamina propria, but not into the detrusor muscle. Initial treatment of NMIBC involves transurethral resection of the bladder tumor (TURBT) for all visible pathology. Further therapy is directed by pathologic stage and grade of the cystoscopically obtained bladder tumor.

According to the tumor-node-metastasis (TNM) classification system, NMIBC can be one of three types: stage Ta (confined to the mucosa), stage T1 (invasive into the submucosa or lamina propria), and carcinoma *in situ* (CIS). Approximately 70% of NMIBCs at initial assessment is Ta, 20% T1, and 10% is CIS[3,4]. Of importance, the intrinsic aggressive potential of this malignancy is strongly associated with tumor grade. By definition, CIS is high grade. Ta and T1 disease may be low or high grade.

## TREATMENT OF HIGH-RISK NMIBC

The initial treatment for all papillary bladder cancers consists of TURBT followed by one immediate dose of chemotherapy for all patients except those with bladder perforation following TURBT or those with muscle-invasive disease[5,6,7,8]. CIS can be found alone or concomitant with papillary disease. When found in conjunction with papillary tumors, all visible tumors should be resected prior to intravesical treatment. Following resection, patients should receive one immediate dose of chemotherapy.

Following initial treatment, the European Association of Urology (EAU) further recommends a second transurethral resection (TUR) for all high-grade papillary NMIBC due to increased recurrence-free and progression-free survival[5,6]. The American Urological Association (AUA) recommends re-resection of both Ta high-grade and T1 lesions if muscularis propria is lacking from the initial specimen, and should be at least considered for those patients with Ta and T1 disease even when muscularis propria is present[6]. Re-resection may allow for increased accuracy of clinical staging.

Patients with high-grade Ta and T1 or CIS NMIBC are at high risk for recurrence and, more importantly, progression. Because of these findings, both the AUA and EUA recommend initial intravesical treatment with bacillus Calmette-Guerin (BCG) followed by maintenance therapy for a minimum of 1 year[5,6]. These recommendations are based on the fact that multiple meta-analyses have shown a decrease in recurrence[9,10,11,12,13,14,15] and progression[14,15] following treatment of NMIBC with BCG.

## SURVEILLANCE

In 2000, the Southwest Oncology Group (SWOG) reported on the use of maintenance therapy following induction with BCG for NMIBC[16]. In order to assess the response to BCG, all patients undergoing treatment underwent transurethral bladder biopsies 3 months after treatment. Many urologists adapted this into practice and routinely perform transurethral bladder biopsies following treatment for high-risk NMIBC. The overall usefulness of biopsying all patients remains under debate.

Multiple reports have suggested that urologists who biopsy all patients after treatment of high-risk NMIBC may be putting their patients through undue risk, and that biopsies should be performed only in select patients based on initial pathology or cystoscopy and cytology following treatment[17,18,19,20,21]. However, a recent report suggests that when cystoscopy and cytology are used alone, up to 37% of patients with active disease would have been overlooked[22]. The increased incidence of disease in the latter two studies was thought to be due to an increased total number of biopsies (both random and directed), as well as prostatic urethral biopsies and isolated renal cytologies.

The identification of bladder cancer in patients with a history of bladder cancer and a normal-appearing urothelium is a well-established phenomenon. In 2003, May et al. prospectively performed six random bladder biopsies on normal-appearing urothelia in 1,033 patients with known bladder cancer[23]. In total, 128 (12.4%) sets of biopsies demonstrated cancer in these areas of the normal-appearing urothelium. Similar results were demonstrated by van der Meijden et al.[24]. Due to the risk of missed diagnosis and delay in future treatment, it is our recommendation that all patients undergoing treatment for high-grade NMIBC undergo a minimum of cystoscopy, bladder cytology, and thorough and consistent bladder biopsies.

Although the use of routine transurethral biopsies following treatment of high-risk NMIBC remains debated, routine cystoscopy does not. Both the AUA and EAU recommend routine cystoscopy following the identification of NMIBC. The AUA does not specifically address the interval or duration of follow-up, nor do they address the use of adjunct urine testing[6]. The EAU recommends that patients with high-risk disease undergo long-term routine surveillance[5]. Patients should have cystoscopy and cytology every 3 months for the first 2 years, every 4 months for the third year, every 6 months for the fourth and fifth years, and annually thereafter. Patients should undergo annual upper tract evaluation as well.

## DESCRIPTION OF TREATMENT FAILURE

The complete response rate to BCG therapy in patients with high-risk NMIBC can be as high as 83.8%[16], however, most patients with high-risk disease suffer from recurrence. It has been estimated that as many as 50% of patients with high-risk disease will suffer from recurrence within 1 year and 90% within 5 years[25]. In the most general sense, any recurrent disease after initiation of BCG therapy can be referred to as “BCG failure”. To aide in the description of the type of BCG failure, specific types of BCG failure should be recognized and include BCG refractory, BCG resistant, BCG relapsing, and BCG intolerant.

BCG-refractory failure refers to an inability to achieve a disease-free state by 6 months after initial BCG therapy, with either maintenance or retreatment at 3 months due to either persistent or rapidly recurrent disease. Any progression in stage, grade, or disease extent by 3 months after the first cycle of BCG would also be included under this description.

BCG-resistant failures describe recurrence or persistence of disease 3 months after an induction cycle. In these cases, the failure is of lesser degree, stage, or grade, and is no longer present at 6 months from BCG retreatment, with or without TUR.

BCG-relapsing failure is the most common type of BCG failure and describes recurrence of disease after achieving a disease-free status by 6 months. BCG-relapsing failure can be further classified into the length of time to recurrence, with early occurring within 12 months of induction, intermediate within 12–24 months, and late occurring >24 months.

BCG-intolerant patients suffer from disease recurrence after receiving an inadequate course of therapy secondary to serious adverse side effects, essentially mandating discontinuation of BCG therapy.

### Options for Post-BCG Failure Treatment

Following recurrence of NMIBC after BCG therapy, treatment options are limited and include repeat BCG treatment, an alternate immunotherapy regimen, chemotherapy, device-assisted therapy, or cystectomy (Table 1). It is important to recognize that all intravesical treatment options at this time are considered investigational.

**TABLE 1**
**Treatments Studied in Patients with BCG Failure**

| Treatment | Medication |
|---|---|
| Immunotherapy | BCG |
| | BCG and IFN-α2b |
| | Mycobacterial cell wall complex |
| | Vicinium |
| Chemotherapy | Gemcitabine |
| | Gemcitabine sequential with mitomycin |
| | Valrubicin |
| | Docetaxel |
| Device-assisted therapies | Electromotive administration of mitomycin |
| | Microwave hyperthermia and mitomycin |
| Cystectomy | |

## *Immunotherapy*

### Repeat BCG Treatment

Although the use of BCG in the treatment of NMIBC was first described by Morales et al. in 1976[26], the mechanism of action is still not entirely understood. Despite the overall effectiveness of BCG, there is still a substantial percentage of patients who do not completely respond to therapy or who will eventually fail therapy. Patients who do not respond to therapy should be offered a second 6-week course of BCG or cystectomy per the guidelines of the AUA and EAU[5,6,27]. Up to 50% of patients treated with a second course of BCG will develop a complete response to therapy[5,27]. About 35% will have a durable (>2-year) response[28]. Further courses of BCG are not recommended because of the reduced chance of success (<20%) coupled with the increased likelihood of tumor progression[28]. It has been shown that after BCG failure, each additional course of BCG carries a 7% actuarial risk of progression[29]. Interestingly, if the recurrence occurs >1 year after BCG response, retreatment is similar to that of BCG-naïve patients[30].

### BCG and Interferon-Alpha2B (IFN-α2b)

IFN-α2b was initially used as a solo intravesical treatment for the treatment of NMIBC. While its efficacy was limited, the side-effect profile was favorable. When used in conjunction with BCG, IFN-α2b was found to synergistically increase the overall immune response[31]. Thus, IFN-α2b was added to low-dose BCG in order to decrease the overall side-effect profile of full-dose BCG and produce similar immune responses due to the synergistic actions of BCG and IFN-α2b. O'Donnell et al. were among the first to evaluate the effectiveness of intravesical IFN-α2b with BCG in 40 patients who had previously failed one or more courses of BCG[32]. Patients received six to eight weekly instillations of low-dose BCG plus 50 million units of IFN-α2b. The rate of disease-free survival was 63 and 53% at 12 and 24 months, respectively. Similar results were reported by Punnen et al. and Lam et al.[33,34].

A recent multi-institutional prospective phase II trial evaluated 1,107 patients treated with BCG and IFN-α2b for NMIBC. Of these patients, 467 had at least one prior BCG failure[35]. Among these patients with prior BCG failure, multivariate analysis revealed that the response to therapy with BCG and IFN-α2b was directly related to the number of prior BCG (but not chemotherapy) failures, as well as the relapse interval between the BCG failures[30].

In all patients with at least one prior BCG failure treated with BCG and IFN-α2b, the complete response was >75%[35]. Over half of the patients with prior BCG were disease free at 2 years, with 45% disease free at 3 years. The results were especially good for CIS single BCG failures, with a 3-year complete response rate of 54%, essentially identical to the 56% found in CIS patients receiving BCG plus IFN the first time. Further evaluation of patients with prior BCG failure revealed that salvage therapy with low-dose BCG and IFN-α2b remained limited for patients who had failed more than one prior BCG treatment. In these patients, the 2-year disease-free survival was only 24% for patients with CIS and 35% for patients with papillary disease[36].

It should be noted that the disease-free survival period in patients treated with BCG and IFN-α2b after prior BCG failures was significantly influenced by the time to failure following previous treatments. In patients with prior BCG refractory disease (<6-month relapse), patients do much worse, but patients relapsing >12 months between treatments have 2-year disease-free rates approaching 60%[30].

### Mycobacterial Cell Wall Complex (MCC)

As mentioned previously, the advent of BCG has changed the landscape in the treatment of high-risk NMIBC. Unfortunately, the use of live bacterial BCG can be associated with serious and even potentially lethal infectious side effects. For this reason, multiple new immunotherapeutic agents have been tested in the treatment of high-risk NMIBC. Specifically, other mycobacterial species and components with anticancer activity toward NMIBC have been investigated[37]. MCC is composed of mycobacterial DNA complexed to the cell wall surface[38]. In 1996, Chin et al. showed that mycobacterial cell wall extract derived from *Mycobacterium phlei* had anticancer activity in murine models[39]. Further studies confirmed the same affect of MCC in human models[38]. Similar to BCG, MCC has been shown to induce apoptosis in cancer cells[37]. Recent studies have shown that both live BCG, as well as *M. bovis* cell wall and DNA extract, can directly induce the release of TNF-related apoptosis-inducing ligand (TRAIL) from neutrophils[40,41,42]. Furthermore TRAIL has been found to induce apoptosis only in cancerous cells and not in normal tissue, thereby explaining the previously results found for both BCG and MCC.

In 2009, Morales et al.[43] treated 55 patients with intravesical MCC. Patients received either 4 or 8 mg of MCC for six consecutive weeks followed by two 3-week maintenance instillations at 12 and 24 weeks. Fifty-one of the patients had CIS with or without concomitant papillary disease. Forty-six patients had previously received and failed BCG treatment. Fifteen of the patients treated with 4 mg MCC and 21 patients treated with 8 mg MCC were evaluable. Complete response rates at 26 weeks were 26.7% (4/15) and 61.9% (13/21), respectively, for the 4- and 8-mg doses. Complete response rates at 18 months were 26.7% (4/15) and 38.1% (8/21), respectively. A phase III trial is currently underway.

### Vicinium

Vicinium is another immune modulator that has recently been used as an intravesical treatment for high-risk patients with CIS refractory or intolerant to BCG. It is a protein comprised of a humanized single-chain variable fragment specific for epithelial cell adhesion molecule and a truncated fragment of *Pseudomonas* exotoxin A[44]. A recently published phase II trial involved 46 patients; 23 received 6 weeks of induction therapy and 23 received 12 weeks. Both groups of patients received maintenance therapy for 3 weeks every 3 months for a maximum of 1 year while tumor free. Of the patients receiving the 6-week induction regimen, 41% (9/22), 32% (6/19), and 16% (3/19) showed complete response at 3, 6, and 12 months, respectively. Of the patients receiving the 12-week induction regimen, 39% (9/23) had complete response at 3 months[45].

## *Chemotherapy*

### Gemcitabine

Gemcitabine is a chemotherapeutic agent with known systemic activity against advanced bladder cancer that has recently been introduced as an intravesicular treatment of NMIBC. Unlike MMC and anthracyclines (discussed later), gemcitabine does not cause contact-tissue injury. Gemcitabine is a deoxycitidine analogue that inhibits DNA synthesis[46,47]. Currently, gemcitabine is being evaluated as an intravesical treatment option mainly in patients who have previously failed BCG therapy. Three initial studies evaluating the use of intravesical gemcitabine in the treatment of BCG-refractory NMIBC have had widely variable results[48,49,50]. Recurrence-free survival for patients with high-risk NMIBC from each respective study was 21% (at 12 months)[49], 56% (at 12 months)[50], and 60% (at 18 months)[48]. As the number of patients treated with CIS increased, the recurrence-free survival decreased. Currently a large multicenter phase II trial looking at the treatment of BCG-refractory NMIBC is under way (SWOG S0353)[51].

More recently, multiple phase III trials comparing gemcitabine with other agents have been published. Addeo et al. reported on the results of a phase III trial comparing the results of gemcitabine vs. mitomycin (MMC) in the treatment of patients with papillary NMIBC who had previously failed intravesical treatment[52]. Of the 109 evaluable patients, 91 had previously failed one BCG treatment. With a median follow-up of 36 months, the recurrence-free survival for the gemcitabine and MMC arms was 72 and 61%, respectively. There was no significant difference with regard to risk of progression. Porena et al. compared the treatment of high-risk NMIBC with gemcitabine or BCG in a randomized controlled trial[53]. A total of

64 patients were treated. At an average follow-up of 44 months, the recurrence-free survival was 71.9 and 46.9% for BCG and gemcitabine treatment arms, respectively. Interestingly the results were much different in a separate randomized controlled study of patients with high-risk NMIBC who received either gemcitabine or BCG after failing one course of BCG[54]. With a median follow-up of approximately 15 months, the recurrence-free survival was 12.5 and 47.5% for the BCG arm and gemcitabine arm, respectively. There was no difference in risk of progression between the two study arms.

Cho et al. recently reported the results of a prospective study comparing the combination of gemcitabine with BCG vs. BCG alone[55]. Patients received either two weekly gemcitabine treatments followed by six weekly BCG treatments or six weekly BCG treatments. Although the recurrence rate was similar between groups, the recurrence-free period was significantly increased in patients treated with gemcitabine and BCG compared to BCG alone (24.1 to 19.8 months).

## Gemcitabine and MMC

While multiagent chemotherapy has become the norm in almost all cases of systemic chemotherapy, the practice is not widely used in the treatment of NMIBC. Initial attempts to use combined or alternating intravesical agents, such as adriamycin and MMC, were frustrated by severe cystitis despite some evidence of enhanced activity[56]. The cystitis associated with adriamycin and MMC administration is thought to be due to their vesicant (tissue irritating) activity, which appeared to act synergistically when used in combination. Because of this and the apparent enhanced activity of multiagent chemotherapies, other combinations of chemotherapeutic agents in which one or both are nonvesicants have recently been evaluated. Two recent studies reported one such combination of immediate sequential gemcitabine (a nonvesicant) followed by MMC. Initial reports found a near 50% recurrence-free rate in 27 BCG refractory patients at 18 months compared to 20% in patients treated with gemcitabine alone in 12 similar patients[57]. Breyer et al. reported similar findings in BCG-refractory or -intolerant patients treated with sequential gemcitabine followed by MMC[58]. In their series of 10 patients, six (60%) remained disease free at a median follow-up of 26.5 months.

## Anthracyclines

Valrubicin is a cytotoxic anthracycline antibiotic used as a chemotherapeutic agent. It acts by inhibiting DNA and RNA synthesis by inhibiting the incorporation of nucleosides into nucleic acids, as well as inhibiting topoisomerase II[47]. Valrubicin is a derivative of doxorubicin, with similar efficacy to the parent compound, but a better toxicity profile, including nonvesicant properties[59].

Valrubicin is approved by the Food and Drug Administration (FDA) for intravesical treatment of BCG-refractory CIS. This approval was based on a phase II study involving 90 patients. This study included patients with CIS ± papillary urothelial cancer after failure of multiple courses of intravesical therapy, including at least one course of BCG. After receiving 800 mg of valrubicin weekly for 6 weeks, 19 patients (21%) had a complete response at 6 months, and seven of these 19 patients (8% of total) had a durable response with a median follow-up of 30 months. An additional 16% of patients had reduction to stage Ta disease, the long-term significance of which is unknown. Notably, all five patients with stage T1 disease (previously resected) plus CIS failed to achieve a complete response at all. Most patients (90%) had mild-to-moderate local bladder symptoms, with urinary frequency in 66%, urinary urgency in 63%, and dysuria in 60%[60].

## Taxanes

Docetaxel is an antimitotic chemotherapeutic agent that has recently been used in the treatment of BCG-refractory NMIBC. Similar to gemcitabine, docetaxel has known systemic activity against advanced bladder cancer and does not cause tissue-contact injury[61]. Docetaxel acts by binding to free tubulin, thereby stabilizing microtubules and inhibiting cell division and leading to cell death[47].

The use of docetaxel as an intravesical treatment for NMIBC was first described in 2006[62]. In this phase I trial, 18 patients with BCG-refractory NMIBC were treated with six weekly treatments of intravesical docetaxel. Complete response was seen in 56% (10/18). Long-term follow-up revealed that 22% (4/18) of patients had durable complete response with a median follow-up of 48.3 months. When maintenance therapy followed a 6-week induction course for 13 BCG refractory patients, 10 (77%) had complete response to therapy and six (46%) remained disease free at a median follow-up of 13 months[63]. Similar long-term results were also seen in a retrospective analysis of 33 patients with BCG-refractory NMIBC receiving intravesical docetaxel[64].

## *Device Assisted Therapy*

### Electromotive Administration of MMC

The use of intravesical MMC alone for salvage treatment following BCG failure has proven to be of minimal benefit. In a study by Malmstrom et al., only four of 21 (19%) patients treated with MMC with one prior BCG failure were disease free at 3 years[65]. Recent advances in the administration of MMC have allowed for MMC to once again be considered for the treatment of BCG-refractory NMIBC. Electromotive drug administration of MMC (eMMC) acts by accelerating ion transport and increasing biological membrane permeability with a subsequent increase in electro-osmesis, and thereby leading to an increased drug accumulation within the bladder wall[66,67]. In 1999, Di Stasi and colleagues showed that electromotive drug administration increased the concentration of MMC four to seven times when compared to passive diffusion in *in vitro* studies[68]. In a randomized trial in 2003, the same group showed that eMMC was equivalent to BCG and significantly better than MMC in the treatment of CIS[69]. In 2008, Sockett et al. reported on the use of eMMC for the treatment of BCG-refractory disease[70]. Thirteen patients with BCG-refractory disease received a 6-week course of weekly eMMC. At 15 months, four of 13 (31%) patients remained recurrence free. Progression was not noted in any of the patients.

### Microwave Hyperthermia

Perhaps a more promising treatment option for patients failing BCG therapy than eMMC involves the administration of MMC by microwave hyperthermia. The addition of thermal energy to the administration of MMC allows for increased cellular permeability of the chemotherapeutic agent, increased DNA cross-linking, and inhibition of DNA repair[71]. Local microwave hyperthermia in conjunction with mitomycin C initially showed promise in the treatment of NMIBC when it was used in the treatment of high-grade Ta or T1 NMIBC. In 24 patients, 62.5% were recurrence free after a mean follow-up of 35.3 months[72].

More recently, this technology has been used in the treatment of high-risk BCG-failure NMIBC. Preliminary results from a multinational prospective European trial using local microwave hyperthermia in intermediate- and high-risk patients were reported in 2004[71]. Of the 90 eligible patients undergoing treatment with local microwave hyperthermia and 40 mg of MMC, 41 (45%) had previously failed BCG treatment. Of these patients, 75% remained disease free at 2 years. Similarly, in 2009, Witjes reported the results of local microwave hyperthermia and the administration of MMC in patients with CIS, 67% (34 of 51) of whom had previously failed BCG[73]. Patients received 40 mg of MMC for an average of 10 treatments. A complete response was appreciated in 45 of 49 (92%) of evaluable patients. Of the 45 complete responders, 23 (51%) remained disease free at an average follow-up of 27 months.

## *Surgical Therapy*

### Cystectomy

If complete response to therapy is not seen by 6 months after beginning initial BCG treatment, cystectomy is the treatment of choice[5,6,27]. Reports from various BCG treatment series involving aggressive NMIBC report a median time to progression averaging 24 months, with rare occurrence before 6 months[74]. Another study showed that tumor response at 6 months was the single most important point for predicting ultimate failure and progression[75]. This corresponds to the use of up to two series of 6-week induction treatments with BCG. Thus, recurrent aggressive disease 6 months after diagnosis or disease persisting after two consecutive courses of BCG therapy are strong indications to consider radical therapy. Furthermore, Herr et al.[76] and Raj et al.[77] report improved long-term survival in patients who received earlier cystectomy (within 2 years) following BCG failure. Because of these findings, both the AUA and EAU advocate for cystectomy following one or two BCG failures; however, further intravesical therapy may be considered.

While all conservative treatments for BCG failure patients remain investigational and uncertain, cystectomy provides for a definitive solution. However, some patients are not candidates for radical surgery due to comorbid medical illness, and others refuse to consider the change in their lifestyle that the surgery entails despite being counseled about the risks. It is for this group of patients that the urologist will exhaust all possibilities to avoid radical surgery. Furthermore, radical cystectomy is associated with 28% morbidity and 2.5% mortality[78]. In a recent article by Shabsigh and colleagues, 64% of patients undergoing cystectomy developed at least one complication within 90 days of surgery[79]. This, among other reasons, could explain why a survey on treatment preferences of NMIBC of 105 practicing U.S. urologists showed that only 19% would treat high-grade Ta-T1 disease that has failed BCG twice with radical therapy[80]. Unfortunately, there have been no prospective randomized trials comparing cystectomy to second-line intravesical therapy for high-risk NMIBC patients. Thus, while alternative therapy continues to be actively developed, cystectomy remains the standard of care for high-risk patients with BCG failure.

## SUMMARY

Current literature suggests that the best treatment for patients with high-risk NMIBC involves BCG therapy. Following treatment, the use of transurethral biopsies to confirm the absence of disease remains under debate. Regardless, close cystoscopic surveillance in these patients is mandatory because of the rate of recurrence and progression associated with high-risk NMIBC. In the presence of BCG failure, further treatment options are complex. Current recommendations include one further course of BCG or cystectomy. In patients who continue to fail conservative treatment and who refuse surgical therapy or are not surgical candidates, treatment options become even more complicated.

Second-line treatment options currently under evaluation for patients that have failed BCG therapy include immunotherapeutic agents, chemotherapeutic agents, and device-assisted therapies (Table 2). The most promising immunotherapies currently available include BCG in combination with IFN-α2b and MCC. Gemcitabine, gemcitabine sequential with mitomycin, and docetaxel also appear to be adequate second-line chemotherapies. Both of the device-assisted therapies mentioned (local microwave hyperthermia and administration of MMC, and electromotive administration of MMC) appear to have a role in the treatment of BCG failures. To date, however, further research is necessary for all secondary treatment options in order to determine which therapy will be the most effective and all conservative treatments should be considered investigational. As it stands, cystectomy remains the standard of care for high-risk patients who have failed BCG therapy.

**TABLE 2**
**Efficacy of Treatments in Patients Failing BCG Therapy**

| | Treatment | Complete Response | Recurrence-Free Survival |
|---|---|---|---|
| BCG Relapsing, Resistant, or Intolerant | | | |
| Immunotherapy | BCG | 50% | 35% (2 years) |
| | BCG and IFN-α2b | 75% | 45% (3 years) |
| | MCC | 62% | 38% (18 months) |
| Chemotherapy | Gemcitabine | | |
| | Ta and T1 only | n/a | 60% (18 months) |
| | All NMIBC | 50% | 21% (1 year) |
| Device-assisted therapy | Microwave and MMC | n/a | 75% (2 years) |
| BCG Refractory | | | |
| Immunotherapy | Vicinium | | |
| | 6-week induction | 41% | 16% (1 year) |
| | 12-week induction | 39% | n/a |
| Chemotherapy | Gemcitabine/MMC | n/a | 60% (2 years) |
| | Valrubicin | 21% | 8% (30 months) |
| | Docetaxel | 61% | 32% (2 years) |
| Device-assisted therapy | eMMC | n/a | 31% (15 months) |
